# Anaphylaxis caused by artisanal honey in a child: a case report

**DOI:** 10.1186/s13256-021-02823-4

**Published:** 2021-05-14

**Authors:** Margherita Di Costanzo, Nicoletta De Paulis, Silvia Peveri, Marcello Montagni, Roberto Berni Canani, Giacomo Biasucci

**Affiliations:** 1 Department of Pediatrics and Neonatology, Guglielmo da Saliceto Hospital, Piacenza, Italy; 2 Department of Translational Medical Science – Pediatric Section, University “Federico II”, Naples, Italy; 3 Department of Allergology, Guglielmo da Saliceto Hospital, Piacenza, Italy; 4 ImmunoNutritionLab - CEINGE Advanced Biotechnologies, University “Federico II”, Naples, Italy; 5 Task Force on Microbiome Studies, University of Naples “Federico II”, Naples, Italy; 6 European Laboratory for the Investigation of Food-Induced Diseases, University of Naples “Federico II”, Naples, Italy

**Keywords:** Food allergy, Honey, Anaphylaxis, Compositae pollen, Case report

## Abstract

**Background:**

Honey is a rare cause of food allergy, especially in children, but it can cause severe systemic allergic reactions. In the pediatric age group, only a few cases have been reported in the literature. Honey allergy may be caused by pollen content or bee-derived proteins. A role for Compositae has been suggested among pollen allergens. Allergology workup of a patient with suspected honey allergy is not well defined. Here we describe a rare case of anaphylaxis in a 5-year-old boy, sensitized to Compositae pollen (ragweed and mugwort), after the ingestion of artisanal honey.

**Case presentation:**

The Slavic patient was referred to our hospital emergency department for generalized urticaria and breathing impairment. All the symptoms occurred approximately 30 minutes after the ingestion of a meal containing salmon and artisanal honey. The allergology workup revealed that a skin prick-by-prick test with the implicated artisanal honey was positive, while a variety of different commercial honey and salmon products yielded negative results. Skin prick test and serum-specific immunoglobulin E (IgE) results were also positive for Compositae pollen (ragweed and mugwort). Patients sensitized to weed pollens who ingest bee products may experience an immediate allergic reaction because of the cross-reaction between weed pollens and Compositae bee product pollen. In this case, primary sensitization may be due to airborne Compositae pollen. Commercial honey is heavily processed due to pasteurization and filtration, which removes most of the pollen. These observations highlight the role of Compositae pollen in the observed allergic reaction and suggest that the different pollen content in the artisanal honey relative to commercial honey was responsible for the allergic reaction in our patient.

**Conclusions:**

This is the first reported pediatric case of honey-induced anaphylaxis in a child under 6 years of age sensitized to Compositae pollen. Pediatricians should be aware of the potential risk of severe allergic reactions upon ingestion of honey and bee products, especially in patients sensitized to weed pollens. To diagnose honey allergy, obtaining a proper clinical history is essential. In addition, skin prick-by-prick tests are helpful, and may represent a simple method to screen for honey allergy in patients sensitized to Compositae pollen, in light of the potential risk.

## Background

Food allergy is a common condition in childhood. Recent studies have suggested that the natural history of food allergy has changed in recent decades, with an increased prevalence, severity of clinical manifestations, and risk of persistence until later ages [1–3]. Honey allergy is a rare form of food allergy, especially in the pediatric age group. So far, only a few pediatric cases have been reported in the literature. Although it is a rare condition, it is important because ingested honey can cause from mild to severe allergic reactions, such as anaphylaxis [4]. Additionally, due to the diverse health benefits of honey and bee products such as propolis and royal jelly [5], increasing honey consumption in health food may increase the incidence of honey-related allergic reactions. Several studies have been performed to identify specific antigenic structures of honey [6]. Honey consists of flower nectar, pollens, and components derived from bees [7]. Honey allergy may be caused by pollen content (especially Compositae pollen) or bee-derived proteins. Also, royal jelly, a secretion of worker honey bee venom, is reported to cause anaphylaxis and asthma exacerbation [8]. Allergology workup of a patient with suspected honey allergy is not well defined. Herein, we report a rare case of anaphylaxis in a patient of pediatric age, caused by an artisanal honey product in a 5-year-old boy sensitized to Compositae pollen.

## Case presentation

The patient was a 5-year-old Slavic boy affected by allergic rhinitis from the age of 4 years (sensitization for dust mite and *Plantago lanceolata*). His past medical history was unremarkable; in particular he had no personal previous history of food allergy or anaphylaxis. He attended nursery school. Familial history revealed that his father had seasonal allergic rhinitis.

The patient was referred to our hospital emergency department by the territorial emergency unit for generalized urticaria and breathing impairment (peripheral oxygen saturation was 93% on ambient air). All the symptoms occurred suddenly 30 minutes after the ingestion of a meal containing salmon and artisanal honey. The intake of other foods/juices, alcohol, or medications was not reported. Salbutamol with inhaler (four inhalations, equivalent to 400 µg) was administered in the ambulance. On admission, the physical examination revealed generalized urticaria and wheeze–bronchospasm in apyrexia. Peripheral oxygen saturation was 97% on ambient air, blood pressure was 100/65 mmHg, heart rate was 120 beats/minute, and respiratory rate was 28 breaths/minute. He was afraid but alert and responsive (Glasgow Coma Scale: 15). Intravenous methylprednisolone (20 mg, equivalent to 1 mg/kg/dose) and chlorpheniramine (5 mg, equivalent to 0.25 mg/kg/dose) were administered, and salbutamol with inhaler (four inhalations, equivalent to 400 µg) was repeated, with progressive and rapid resolution of cutaneous and respiratory symptoms.

The results of routine laboratory analyses on admission were within the normal range (see Table 1). Tryptase serum levels and specific food IgE tests were performed for egg (0.31 kUA/L), milk (0.67 kUA/L), shrimp (0.08 kUA/L), cod (0.11 kUA/L), gluten (0.27 kUA/L), lipid transfer protein (LTP) Pru p 3 (0.12 kUA/L), soy (0.72 kUA/L), grass pollen (16.5 kUA/L), *Dermatophagoides pteronyssinus* (3.67 kUA/L), ragweed (7.93 kUA/L), and mugwort (35.3 kUA/L), using the ImmunoCAP (Thermo Fisher Scientific, Sweden). Levels ≥ 0.35 kUA/L were considered positive.

**Table 1:** Routine laboratory findings on admission

White blood cells (× 10^3^/µL)	10.99
Red blood cells (× 10^6^/µL)	4.5
Platelets (× 10^3^/µL)	287
Hemoglobin (g/dL)	12
Hematocrit (%)	34.4
Neutrophils (%)	49.3
Lymphocytes (%)	39.7
Monocytes (%)	7.5
Eosinophils (%)	3.1
Basophils (%)	0.4
C-reactive protein (mg/dL)	0.05
Glycemia (mg/dL)	123
Urea (mg/dL)	27
Creatinine (mg/dL)	0.26
Sodium (mEq/L)	136
Potassium (mEq/L)	3.8
Chlorine (mEq/L)	107
Calcium (mg/dL)	9.27
Aspartate aminotransferase (U/L)	39
Alanine aminotransferase (U/L)	15

The reaction tryptase level was 6.63 µg/L (normal values <11 µg/L), whereas the post-reaction level, detected 24 hours after the allergic event, was 2.04 µg/L. In the pediatric age group, tryptase reaction levels exceeding a threshold level of 2 ng/mL + 1.2 × (post-reaction tryptase level) may be very useful in establishing a diagnosis of anaphylaxis [9]. In our patient, the reaction tryptase level exceeded the threshold level of 4.18 µg/L.

The test for serum IgE antibodies to bee venom yielded a weak positive result (0.65 kUA/L). However, his personal history was negative for bee stings, and sensitization to hymenoptera venom is frequently found in atopic and non-atopic subjects. In particular, in atopic patients, a high sensitization rate has been observed and could partially be explained by cross-sensitization between pollen and hymenoptera venom due to specific IgE to cross-reactive carbohydrate determinants [10].

The patient was discharged after 24 hours of clinical observation in good condition and without drug therapy. At the time of hospital discharge, an allergology follow-up was scheduled for 2 weeks later. Skin prick tests with ragweed (*Ambrosia artemisiifolia*) and mugwort (*Artemisia vulgaris*) were positive, while prick-by-prick tests with salmon and peanut were negative. As for honey, prick-by-prick test with the mixture of flower artisanal honey that the patient consumed before allergic reaction was positive. On the contrary, prick-by-prick test with a commercial flower honey mixture, Millefiori (a kind of honey frequently consumed in our country, obtained from foraging on Compositae), was negative. Positive (histamine) and negative (saline solution) controls were included. The reactions were read after 15 minutes and were positive if there was a wheal 3 mm or greater. Based on the patient’s clinical history and allergy test results, we made a diagnosis of anaphylaxis induced by honey. An oral provocation test was not performed because of the personal recent history of anaphylaxis. The patient was informed of the honey allergy and the importance of honey avoidance. An adrenaline auto-injection kit (0.15 mg) was prescribed and the patient was instructed on its usage. After a year of follow-up, he had been able to avoid honey and remained asymptomatic.

## Discussion and conclusions

We report the first pediatric case of honey-induced anaphylaxis in a child under 6 years of age sensitized to Compositae pollen reported in the literature.

Anaphylaxis is a life-threatening allergic reaction, and it is important to confirm the etiology to prevent recurrence. Severe allergic reactions caused by honey are rare. In the literature, honey allergy is usually attributed to the pollen content. Honey contains significant amounts of pollens, and this accounts for the role of pollen allergens in allergic reactions to honey. The importance of pollen allergens is confirmed by their identification in immunoblotting studies [6]. However, the pollen content of honey depends on the location and the season when pollens are collected by honey bees [11]. The most common pollens responsible for the reactions are assumed to be those of the Compositae family.

Our patient had no previous history of anaphylaxis after a bee sting or food allergy. He had a personal clinical history of allergic rhinitis from the age of 4 years, with skin prick tests and serum-specific IgE initially positive to dust mite and *Plantago lanceolata*. After the allergic reaction with honey ingestion, the allergology workup (skin prick test and serum-specific IgE) also yielded positive results for Compositae pollen: ragweed (*Ambrosia artemisiifolia*) and mugwort (*Artemisia vulgaris*).

Moreover, we observed that the prick-by-prick test yielded a positive result for artisanal honey and negative result for commercial honey. Commercial honey is heavily processed due to pasteurization and filtration, which removes most of the pollen. These observations highlight the role of Compositae pollen in the observed allergic reaction and suggest that the different pollen content in the artisanal honey relative to the commercial honey was responsible for the allergic reaction in our patient.

Patients sensitized to weed pollens who ingest bee products (honey, royal jelly, bee pollen) may experience an immediate allergic reaction because of the cross-reaction between weed pollens and Compositae bee product pollen [12]. In this case, primary sensitization may be due to airborne Compositae pollen.

It is known that patients with pollinosis may display clinical characteristics caused by allergy to certain fruits and vegetables, but the mean age for the beginning of allergic symptoms is usually adulthood, and clinical manifestations are generally mild such as oral allergy syndrome [13].

To the best of our knowledge, this is the first reported pediatric case of honey-induced anaphylaxis in a child under 6 years of age sensitized to Compositae pollen. With regard to bee products in pediatric patients, Martín-Muñoz *et al.* described a 4-year-old boy with allergic symptoms, but without anaphylaxis, immediately following ingestion of bee pollen as a food supplement [14]. Another case of honey-induced anaphylaxis was described in a 14-month-old boy, but in this case the authors found no sensitivity to pollens or bee venoms [15].

In conclusion, despite being a rarely observed condition, honey allergy has serious consequences, even in childhood. Allergic reactions to honey can be related to many factors, including pollens. Pediatricians should be aware of the potential risk of severe allergic reactions upon ingestion of honey and bee products, especially in patients sensitized to weed pollens. To diagnose honey allergy, obtaining a proper clinical history is essential. In addition, skin prick-by-prick tests are helpful, and may represent a simple method to screen for honey allergy in patients sensitized to Compositae pollen, in light of the potential risk.

## Data Availability

The data sets used and/or analyzed during the current study are available from the corresponding author on reasonable request.
